# Supplementary material for: A dynamic basal complex modulates mammalian sperm movement
Source: Nat Commun. 2021 Jun 21;12:3808. doi: 10.1038/s41467-021-24011-0 (PMC8217517; doi:10.1038/s41467-021-24011-0)
Supplement: Supplementary file 1 — Supplementary Information [file 41467_2021_24011_MOESM1_ESM.pdf]

Supplementary Figures

Supplementary Fig. 1

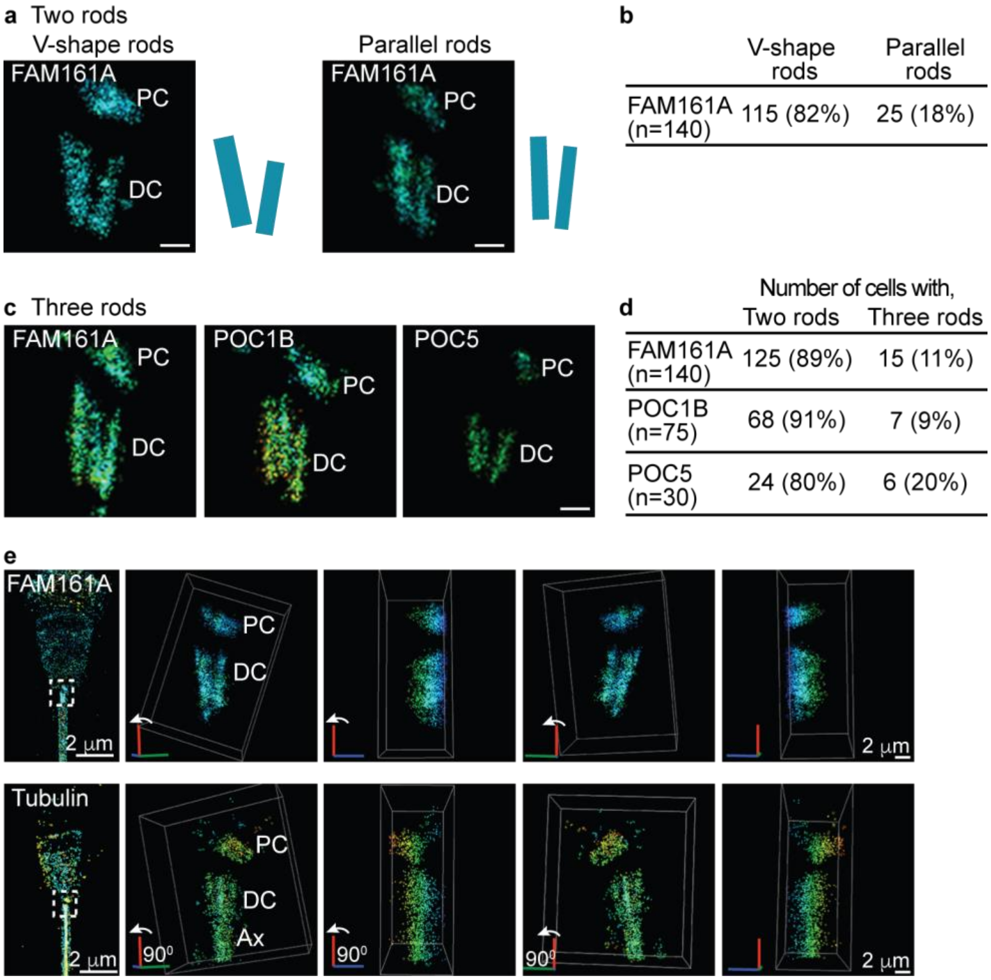

Supplementary Fig. 2

a

PC microtubules diameter	STORM	n	Cryo-ET	n	P
Base (nm)	252±23	27	253±16	20	0.9
Tip (nm)	236±31	27	229±19	20	0.5
Axoneme (nm)	211±27	27			

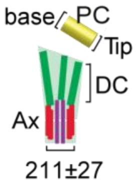

b

	Rods POC5	n	Bars	n	P
Angle	12±3	30	5±6	20	<0.0001

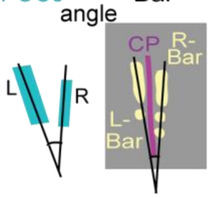

c

	Rods POC5	n	Bars	n	P
The external edge to edge gap(nm)	280±31	30	187±27	20	<0.0001

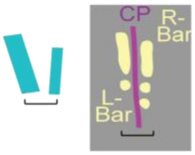

d

	L-Bars	n	R-Bars	n	P
Number of plates	1.5±0.61	20	2.2±0.77	20	0.003
Overall length (nm)	435±60	20	500±124	20	0.05
Distance to same side microtubule (nm)	171±26	13	105±25	13	<0.0001

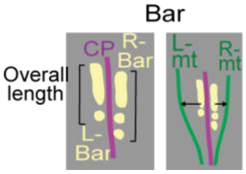

e

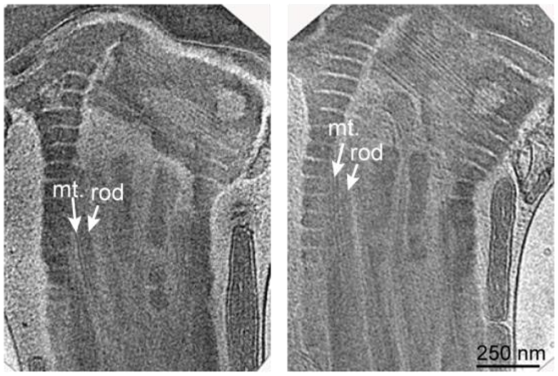

f Statistical analysis (p values)

rods/mts.	left rod vs right rod length	left rod vs right rod width
POC5	1E-05	4E-06
POC1B	3E-16	3E-16
FAM161A	2E-07	6E-08
Tubulin	0.02	

Supplementary Fig. 3

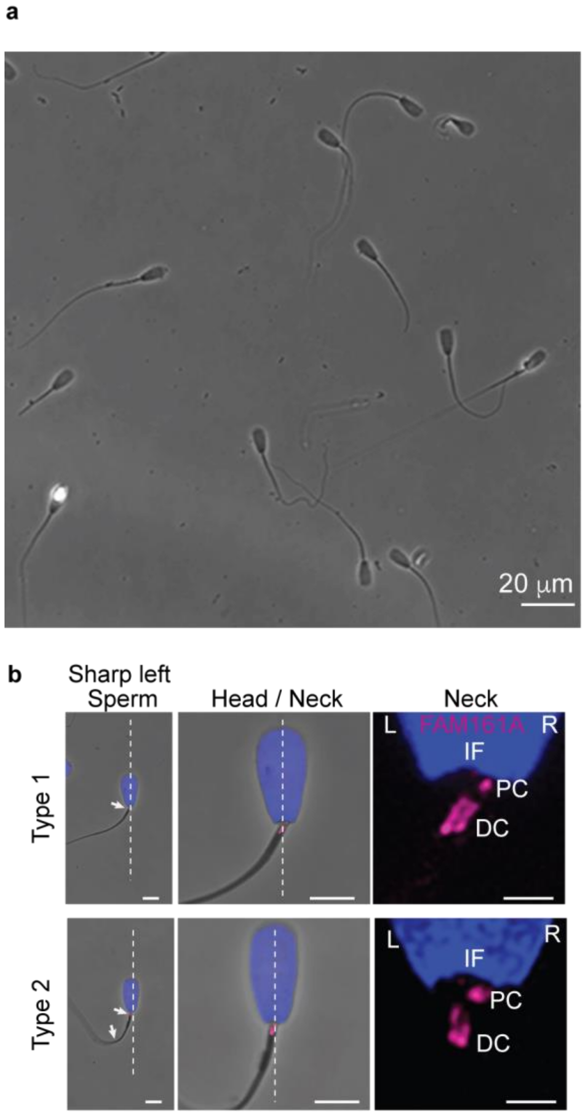

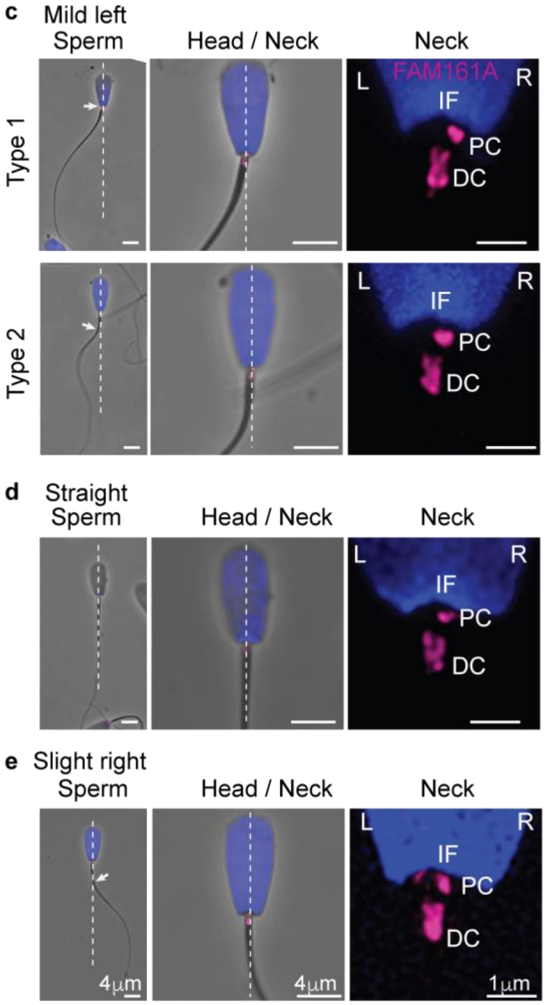

f

Exp #, n	Sharp left	Mild left	Straight	Slight right	Sharp right
1, 80	11	23	29	17	0
2, 51	8	13	20	10	0
3, 69	9	23	25	12	0
4, 48	9	15	16	8	0
% of total, 248	15	30	36	19	0

*

****

Supplementary Fig. 4

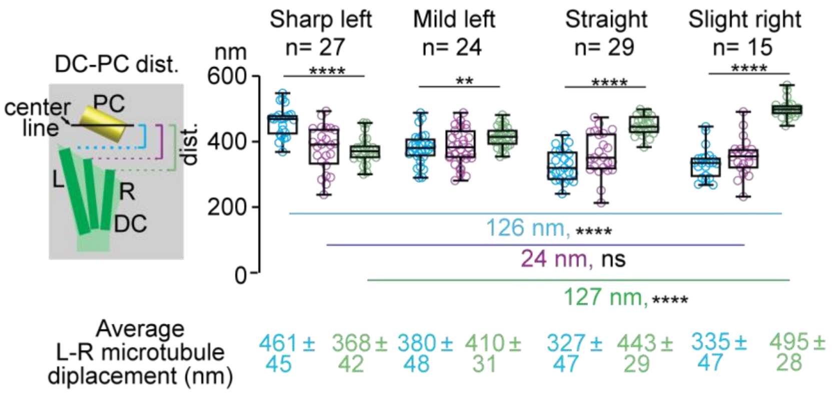

Supplementary Fig. 5

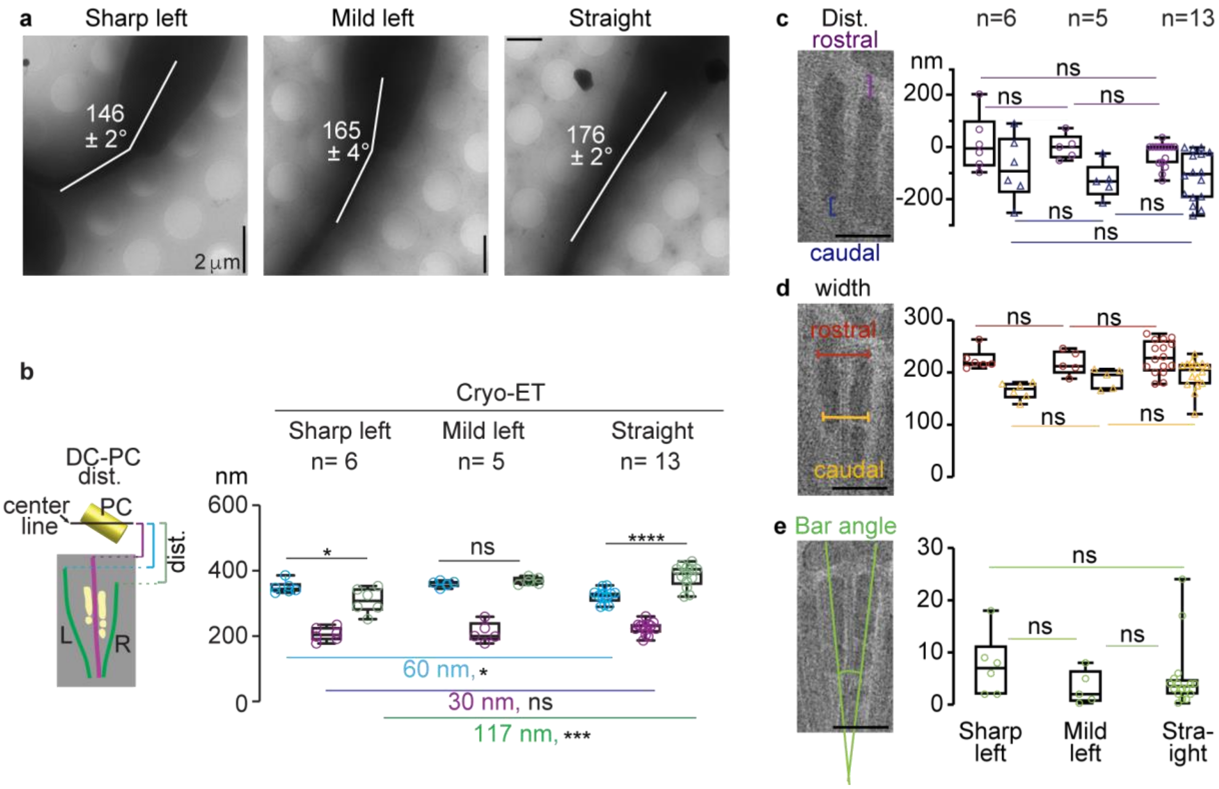

Supplementary Fig. 6

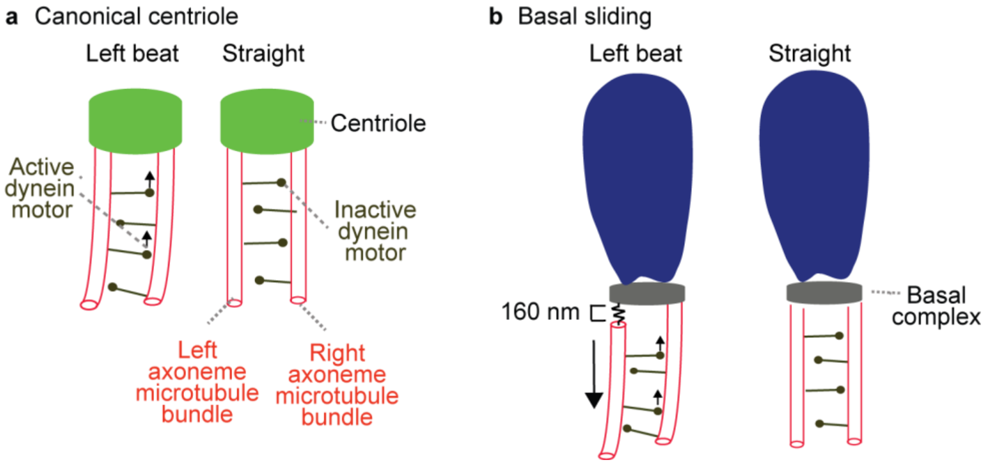

Supplementary Fig. 7

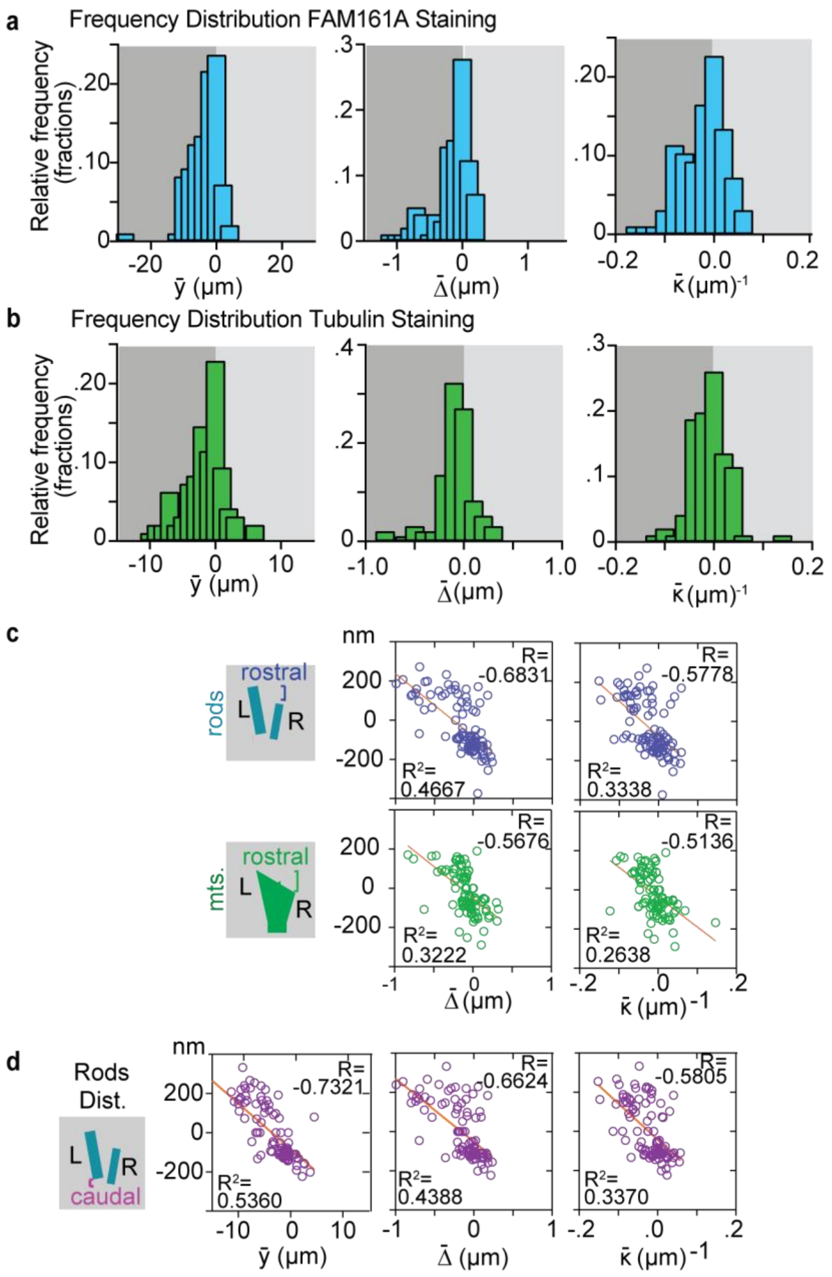

Supplementary Fig. 8

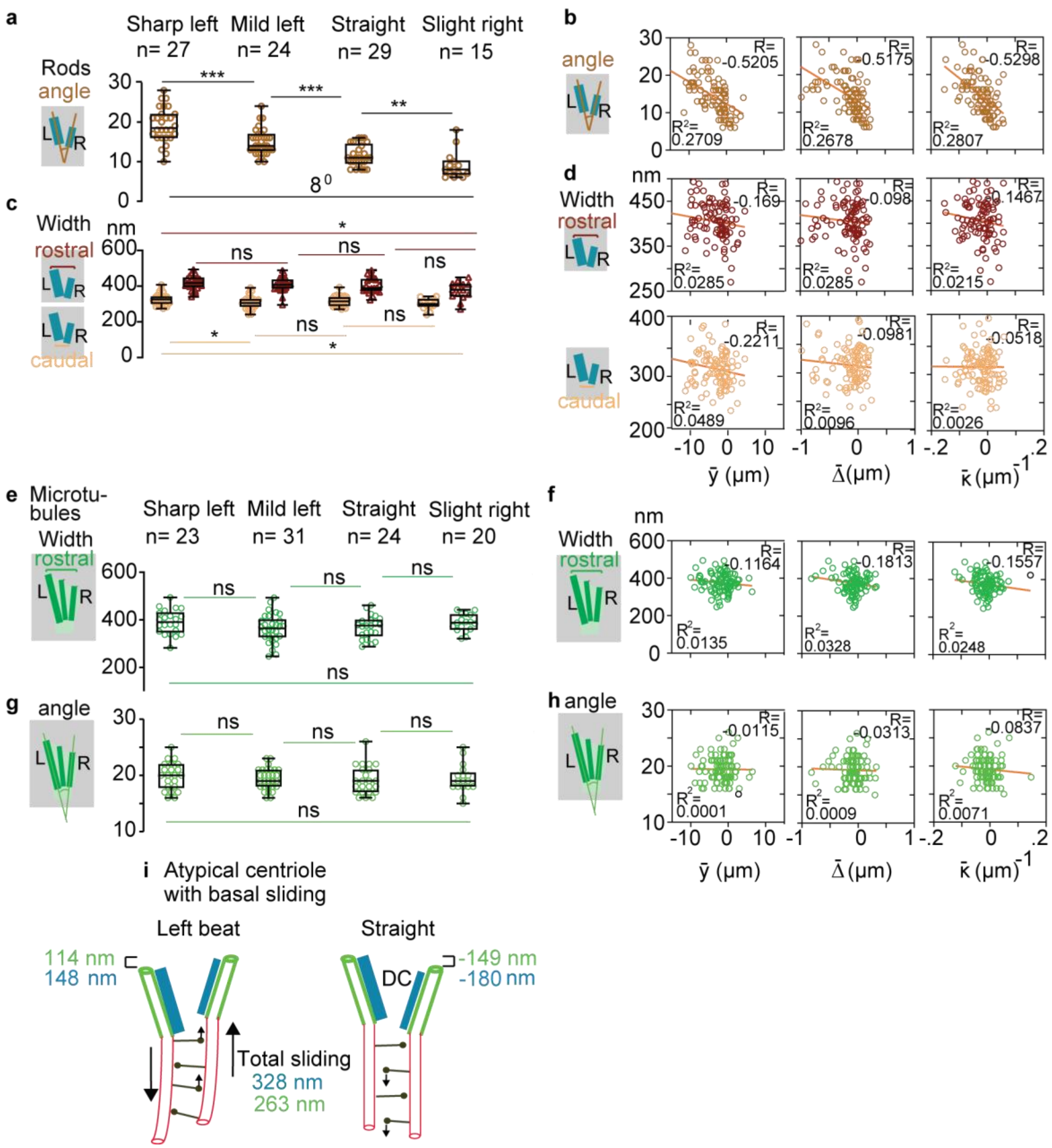

Supplementary Fig. 9

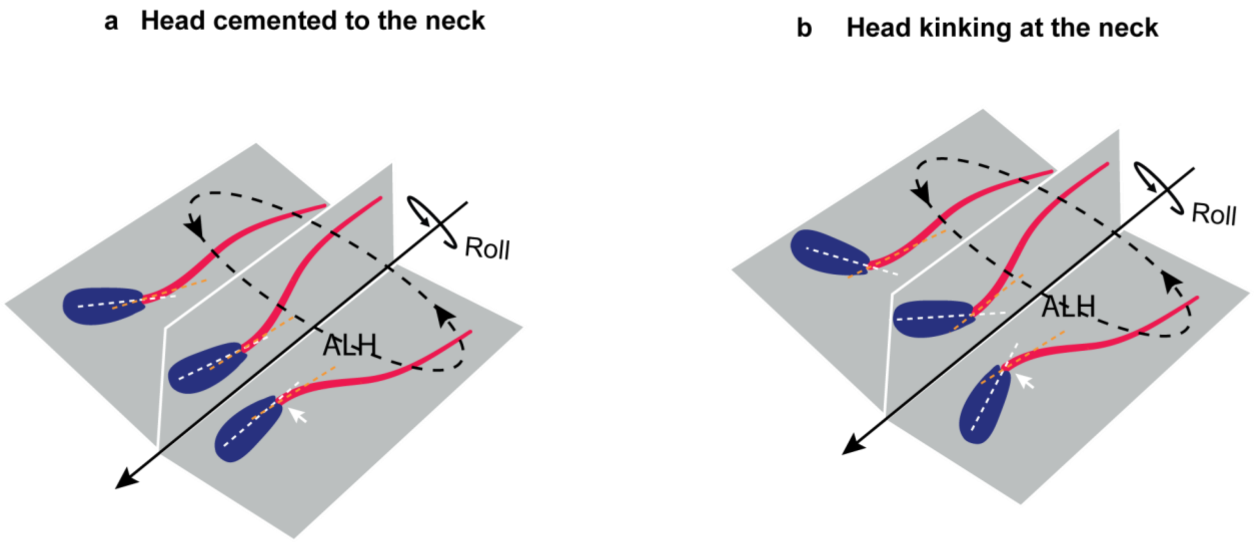

Supplementary Fig. 10

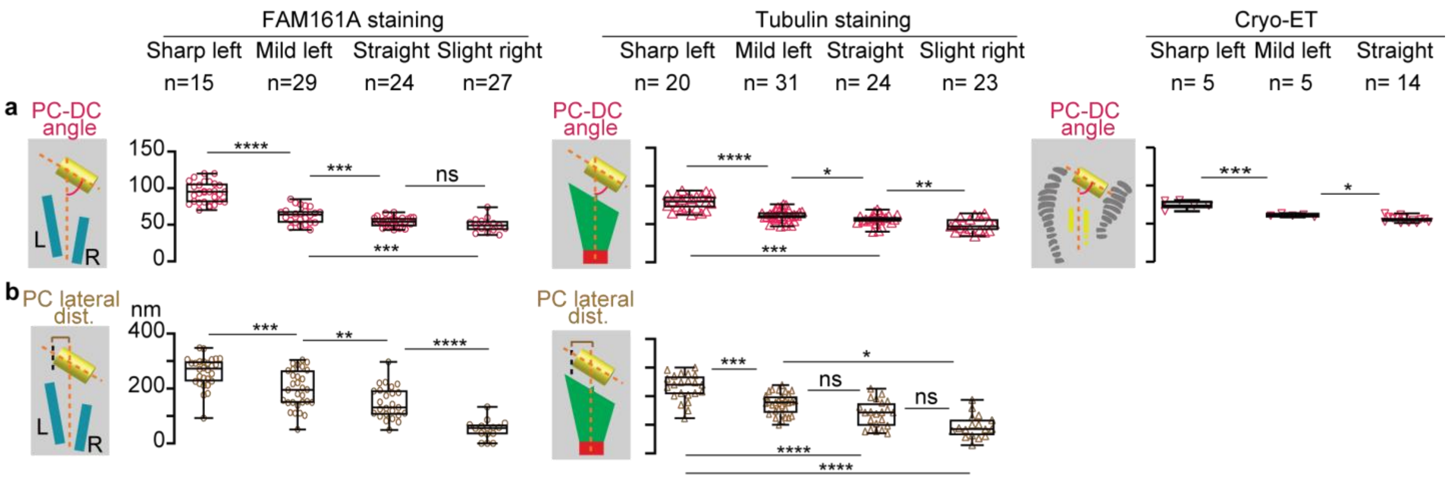

Supplementary Fig. 11

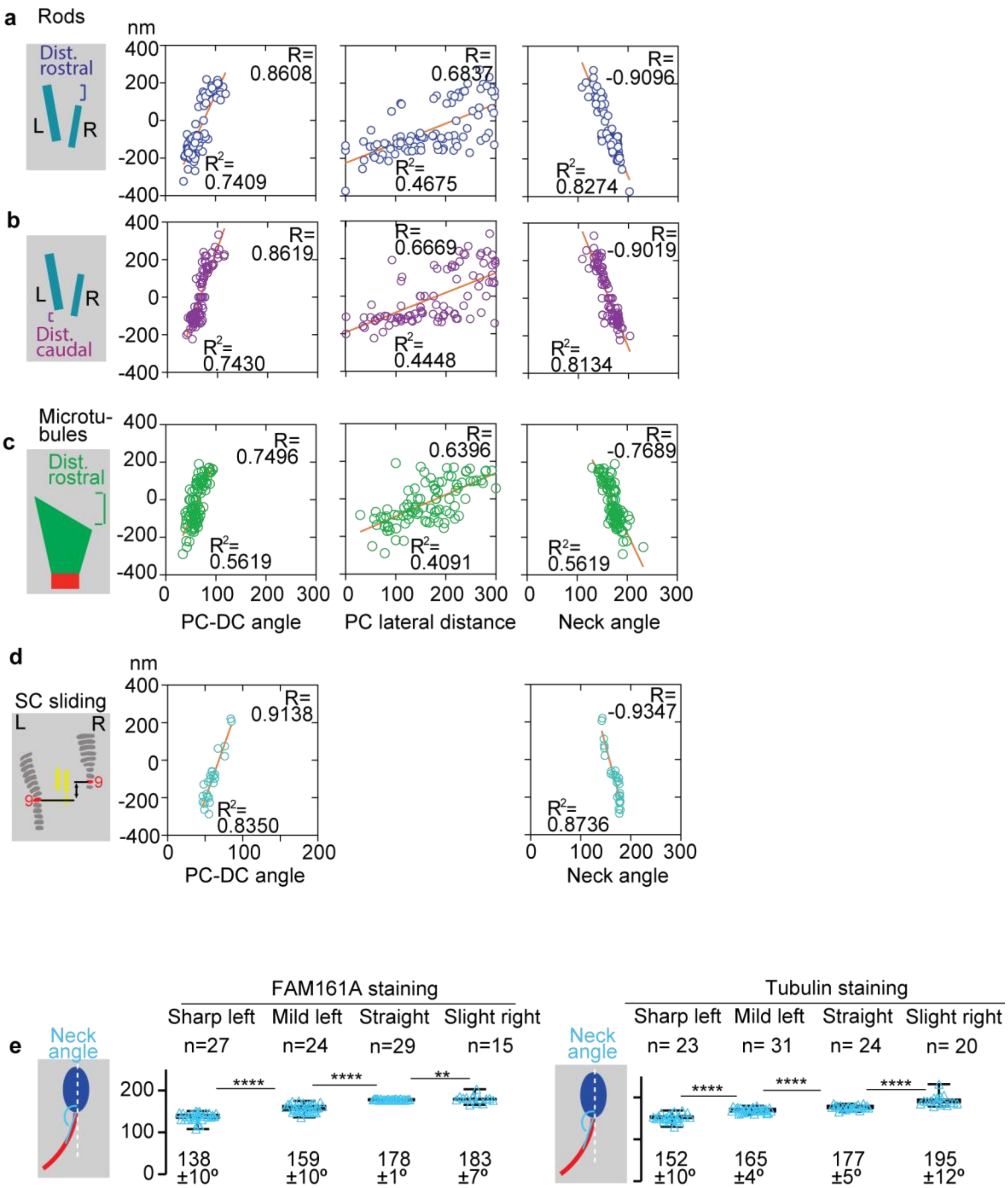

Supplementary Fig. 12

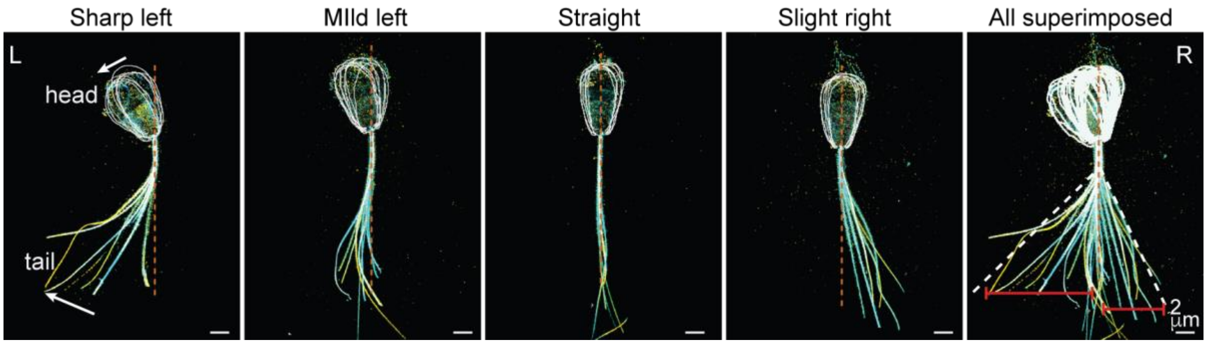

Supplementary Fig. 13

Structure	variable #	a	Mov	Sta	b	Mov	Mix	Sta	c	Mov	Sta
Head	1	Head-neck angle	.90		Head-neck angle	.90			Head-neck angle	.95	
Neck PC	2	PC-DC angle	.83		PC-DC angle	.88			PC-DC angle	.96	
	3	PC lateral distance (R)	.76		PC lateral distance (R)		.48	.60			
	4	PC lateral distance (L)	.77		PC lateral distance (L)	.80					
Neck DC Rods	5	DC rod rostral distance	.89								
	6	DC rod caudal distance	.88								
	7	L-R rod angle	.67								
	8	DC rod rostral width		.91							
	9	DC rod caudal width		.89							
Neck DC Mts	10				DC Mts rostral distance	.86					
	11				L-R Mts angle		.79				
	12				DC Mts rostral width			.87			
Neck DC Bars	13								Bar cauda width	.62	.56
	14								Bar rostral width		.88
	15								Bar rostral distance		.60
	16								Bar caudal distance		.55
	17								L-R bar angle		.69
Neck SCs	18								SCs distance	.95	
Tail	19	Interfilament sliding (Δ)	.85		Interfilament sliding (Δ)	.74					
	20	Curvature (k)	.78		Curvature (k)	.65	.46				
	21	Amplitude (y)	.89		Amplitude (y)	.92					

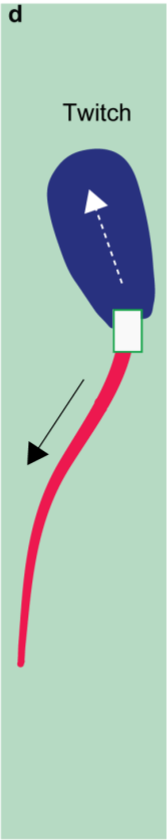

Supplementary figure legend

Supplementary Fig. 1: Bovine sperm DC predominantly has two rods arranged in a V shape.
a Representative images of bovine sperm necks stained for FAM161A showing two rods with either V-shape or nearly parallel confirmations (≤ 8^0). Scale bars: 250 nm. **b** Occurrence distribution of V-shape or parallel rod conformations as assessed by FAM161A staining. **c** Representative images of bovine sperm necks stained for different rod proteins showing three rods. Scale bars: 250 nm. **d** Occurrence distribution of sperm with either two rods or three rods. **e** DC rods (upper panel) and microtubules (lower panel) are shown at four viewing angles. The left two panels are low magnification images of sperm cells and inset zooming neck. Three panels on the right side are each with 90^0 rotations in an anticlockwise direction. Rotation is shown at the lower-left corner with X (green line), Y (red line), and Z-axis (blue line). All experiments were repeated three times independently with similar results. Labels are same throughout the paper, PC, Proximal centriole; DC, distal centriole; Ax, axoneme. Source data are provided as a source data file.

Supplementary Fig. 2: Rods and bars show distinct organization inside the DC.

a Measurement of proximal centriole (PC) diameter based on STORM imaging with tubulin staining and measurement of PC microtubule diameter based on cryo-ET. **b** Comparison of angles between POC5 L-R rods and bars. **c** POC5 rod and bar width at the caudal end. **d** Number of plates in left (L) and right (R) bars, overall length of L-R bars, and L-R bar distance from respective side microtubules. L-mt, Left microtubules; R-mt, Right microtubules; L-Bar, left bars; R-Bar, right bar. All measurements are shown as mean ± sd nm. **e** Cryo-ET images marking doublets and putative location of rods in DC. An elongated electron density is observed along the inner surface of the lateral DC doublet microtubules. This density is located peripheral to the bars and corresponds to the rods' expected location based on STORM measurements. We marked this location on pictures shown in Fig. 2f and a new cell. mt., microtubules**.** **f** Statistical analysis comparing left and right rods stained for various proteins. Statistical analysis used to determine *p* values is an unpaired, two-tailed *t*-test. All measurements are shown in Fig. 2b–e. Experiments were repeated 2-3 times independently with similar results. Source data are provided as Source Data File.

Supplementary Fig. 3: Sperm tail beating is biased towards the left side.

a Low magnification phase image with varying types of sperm tail beating in snap-frozen sperm cells. **b–e** Images showing sharp left sperm cells **(b)**, mild left **(c)**, straight **(d)**, and slight right cells **(e)**. The first panel shows a low magnification whole sperm cell with a straight head and direction of tail beating relative to the head midline. The second panel shows a zoom-in of the head and neck, and the third panel shows a zoom-in of the neck emphasizing implantation fossa and PC at the right side of sperm cells. The two types in (b) and (c) are subgroups of sperm characterized by tail bending location. The white arrowhead marks the tail bending position. The white dotted line marks the head-neck midline. The arrow marks the location of tail bending. L: left, R: right, IF: Implantation fossa. **f** Table summarizing four experiments, each carried out with 48-80 sperm, (n) that found the percentage of sperm in each category of tail curvature. Experiment was repeated three times independently with similar results. Statistical analysis used to determine *p* values is an unpaired, two-tailed *t*-test. **P*<0.05, *****P*<0.0001. Source data are provided as Source Data File.

Supplementary Fig. 4: DC central microtubules move less than left- and right-side microtubules.

Measurements showing the distance between the PC central line (marked by black line) and DC left-, central-, and right side microtubules based on 3D-STORM imaging. The average L-R microtubule displacement shows the mean ± sd distance (dist.) between the PC centerline and L-R microtubules in four different groups of sperm cells. Data are presented as box and whisker plot, where upper and lower bounds show interquartile range, line within the box shows median, and whiskers show minimum and maximum data points. The experiment was repeated three times independently with similar results. An unpaired, two-tailed *t*-test was used for determining *p* values. ***P*<0.01, *****P*<0.0001; ns, not significant. Source data are provided as Source Data File.

Supplementary Fig 5: Bar positions are relatively stable during sperm tail beating.

a Low-magnification cryo-EM projection images of bovine sperm, showing head-neck angles in three different groups of cells. **b** Measurements showing the distance (dist.) between PC centerline and left-, central-, and right-side microtubules, based on cryo-ET imaging. Statistical analysis used is an unpaired, two-tailed *t*-test. **P*<0.05, ****P*<0.001, *****P*<0.0001, ns, not significant. **c–e** No significant difference is detected between sharp left and straight sperm cells, which includes measurements of the rostral and caudal distance of bars **(c)**, the width between the bars **(d)**, and the angle between the bars **(e)**. The measurement scheme is shown in the figures or schematics on the left side of each graph. The Y-axis shows various measurements, and the X-axis shows the various groups of cells, categorized based on tail bending. Data are presented as box and whisker plot, where upper and lower bounds show interquartile range, line within the box shows median, and whiskers show minimum and maximum data points. Statistical analysis used to determine *p* values is an unpaired, two-tailed *t*-test. All experiments in this figure are repeated twice independently with similar results. Scale bars **(c-e)**, 0.25 µm. ns, not significant. Source data are provided as Source Data File.

Supplementary Fig. 6: Basal sliding hypothesis.

a Schematic representation of the classical model of axoneme sliding movement without sliding at the base upon flagellar bending; i.e., the microtubules are cemented at the base. **b** Schematic representation of the basal sliding model, including predicted basal sliding displacement (black arrow) of 160 nm in bovine sperm from the most widely accepted motor-control hypothesis [1]. In this model, the basal complex (grey region) is considered a rigid structure that anchors the tail firmly to the head while allowing microtubule sliding at the basal end with little compliance allowed by the segmented columns.

Supplementary Fig. 7: Tail beating is biased to the left and is correlated with the DC's internal sliding.

a–b Histogram showing distributions of average amplitude, $\bar{y}$ (µm), of tail beating, microtubule sliding, $\bar{\Delta}$(µm), and curvatures, $\bar{\kappa}$ (µm) $^{-1}$, measured with FAM161A **(a)** and tubulin **(b)** staining. The dark and light shadow depicts the left and right sides of the tail beating with all three variables ($\bar{\Delta}$, $\bar{\kappa}$, $\bar{y}$). **c** Correlation analysis between the rod and microtubule rostral distance during sperm tail beating and tail variables ($\bar{\kappa}$, $\bar{y}$). **d** Correlation analysis between the rod distance at caudal end during sperm tail beating and all three tail variables. Statistics of all correlation analyses is *****P*<0.0001. Total, n=97 sperm cells with FAM161A staining and n=91 with tubulin staining under three independent experiments were analyzed. Source data are provided as Source Data File.

Supplementary Fig. 8: The geometric clutch hypothesis may not be acting during sperm tail beating.
Correlation analysis between the microtubule angle and the flagellar waveform (**h**), indicating that "geometric clutch" effects may be absent at the DC [2]. **a-b** Measurement of rod angle during sperm tail beating **(a)** and correlation analysis with three tail variables ($\bar{\Delta}$, $\bar{\kappa}$, $\bar{y}$) **(b)**. Statistics of correlation analysis, *****p*<0.0001. **c-d** Measurement of rod rostral and caudal width **(c)** during sperm tail beating and correlation analysis with tail parameters **(d)**. *P<0.05; ns, not significant. **e-h** Measurement of microtubule rostral width **(e)** and correlation analysis with tail variables **(f)**. Microtubular angle measurement **(g)** and correlation analysis **(h). i** Schematic representation of the dynamic basal complex, showing its constituent microtubules and rods sliding bidirectionally at the base end between sharp left to slight right beating. Data in Fig. 8a, 8c, 8e, and 8g are presented as box and whisker plot, where upper and lower bounds show interquartile range, line within the box shows median, and whiskers show minimum and maximum data points. Statistical analysis used to determine *p* values is an unpaired, two-tailed *t*-test. Correlation analysis in Fig **7d**, **f**, and **h** are not significant (ns). Source data are provided as Source Data File.

Supplementary Fig. 9: Types of sperm head motion: rolling, lateral displacement, and kinking.

A schematic depiction of sperm movement with a head cemented to the neck (**a**) and sperm movement with a head movement relative to the neck (**b**). The spiral movement of spermatozoa swimming through a medium

includes two previously described types of movements – rolling and lateral displacement (**a**) – as well as a third type that we have discovered in this study: kinking (**b**). Rolling is the rotational movement of spermatozoa around their long axis [3-5]. The rolling is due to the three-dimensional component of the beat of the flagellum. Lateral displacement is the two-dimensional appearance of circular motion of spermatozoa along their swimming path. Kinking is the back-and-forth motion of the spermatozoan head during sperm tail beating. ALH, amplitude of lateral head displacement. White and orange dotted lines show head and neck midlines, respectively. Arrow marks the kink at the neck.

Supplementary Fig. 10: PC position changes during sperm tail beating.

a PC angle relative to the neck midline as the sperm tail bends from sharp left to right, shown based on STORM (left two panels) with FAM161A and tubulin staining and Cryo-ET (right panel). The angle between PC and neck midline was measured. **b** PC lateral position relative to the neck midline as the sperm tail bends from sharp left to right, shown based on STORM imaging with FAM161A and tubulin staining. The lateral distance was measured between neck midline and the left edge of the PC. Data are presented as box and whisker plot, where upper and lower bounds show interquartile range, line within the box shows median, and whiskers show minimum and maximum data points. Statistical analysis used to determine *p* values is an unpaired, two-tailed *t*-test. *$P<0.05$, **$P<0.01$,***$P<0.001$,****$P<0.0001$, ns, not significant.

Supplementary Fig. 11: DC and SC sliding correlate with PC and head position.

a–d Correlation analysis of change in PC-DC angle, PC lateral position, and head-neck angle with rod sliding at the rostral end **(a)**, rod sliding at the caudal end **(b)**, microtubule sliding at the rostral end **(c)**, and SC sliding **(d)**. Statistics of correlation analysis, ****$P<0.0001$. **e** Change in head-neck angle as the sperm tail bends from sharp left to right, shown based on STORM with FAM161A (left) and tubulin (right) staining. The head-neck angle of each group is shown as mean ± sd below the graph. Statistical analysis used to determine *p* values is an unpaired, two-tailed *t*-test. **$P<0.01$,****$P<0.0001$.

Supplementary Fig. 12: The head kinks sharply to the left when the tail bends to the left.

Multiple cells from four different groups were observed in 3D-STORM with tubulin staining. The staining was enhanced to visualize the head of the cells. The head is outlined with a white line. Multiple cells with a range of curvatures were selected for each group. The cells were aligned at the neck and superimposed together. The white arrow shows direction of head and tail bending. Orange dotted line marks the neck midline. The neck midline is not co-aligned with head and flagellum in sharp left cells. The red line in all superimposed cells marks the tail beating amplitude on left and right sides relative to head

Supplementary Fig. 13: The 21 sperm variables obtained from 3 data sets analyzed by Exploratory Factor Analysis (EFA).

Three latent factors were identified: the tail-to-head coordinated movement (Mov) factor, the no movement of DC center and width (Sta) factor, and a mixture of the two (Mix) factors, suggesting a slight movement of some parts. Factor loadings are provided for each factor. Note that all factor loadings for both the Mov and Sta factors are above 0.5. Loadings (correlations) for each factor between variables are shown in columns marked as Mov, Mix, and Sta. **a** A data set of 97 sperm cells obtained by immunostaining for FAM161A. **b** A data set obtained by immunostaining for tubulin and which included 93 sperm cells. **c** A data set obtained by cryo-EM and which included 21 sperm cells. **d** A sperm cell representing its various parts. EFA analysis shows coordinated movements of tail beating, neck deformation, and head kinking, resulting in a twitching movement. Source data are provided as Source Data File.

Supplementary table

Antibody	Confocal dilution	STORM dilution	Source and catalog number
FAM161A	1:300	1:100	Sigma Aldrich, #HPA032119
WDR90	1:100	NA	Sigma Aldrich, #HPA061785
POC5	NA	1:80	Thermo Fisher Scientific, # PA5-24308
POC1B	NA	1:80	Thermo Fisher Scientific, # PA5-24495
CETN1 Clone 2A6	1:20	NA	Santa Cruz, # sc-293494
Beta Tubulin (E7)	NA	1:20	DSHB, AB_2315513
Anti-rabbitA647	1:300	1:100	Jackson Immunoresearch, #711-605-152
Anti-mouse647	1:300	1:100	Jackson Immunoresearch, #715-605-150
Anit-mouse488	1:200	NA	Jackson Immunoresearch,# 715-545-150

Supplementary table 1: Antibodies used in this study. NA – not applicable. Note that as was reported previously [31] the WDR90 staining is weak and unsuccessful in STORM imaging of bovine and human sperm even with antibody dilution used at 1/20.

Supplementary references

1 Riedel-Kruse, I. H., Hilfinger, A., Howard, J. & Jülicher, F. How molecular motors shape the flagellar beat. *HFSP journal* **1**, 192-208, doi:10.2976/1.2773861 (2007).

2 Lindemann, C. B. A Geometric Clutch Hypothesis to Explain Oscillations of the Axoneme of Cilia and Flagella. *Journal of Theoretical Biology* **168**, 175-189, doi:10.1006/jtbi.1994.1097 (1994).

3 Ishijima, S., Hamaguchi, M. S., Naruse, M., Ishijima, S. A. & Hamaguchi, Y. Rotational movement of a spermatozoon around its long axis. *J Exp Biol* **163**, 15-31, doi:10.1242/jeb.163.1.15 (1992).

4 David, G., Serres, C. & Jouannet, P. Kinematics of Human-Spermatozoa. *Gamete Research* **4**, 83-95, doi:DOI 10.1002/mrd.1120040202 (1981).

5 Mortimer, S. A critical review of the physiological importance and analysis of sperm movement in mammals. *Human Reproduction Update* **3**, 403-439, doi:10.1093/humupd/3.5.403 (1997).
