# Supplementary material for: A dynamic basal complex modulates mammalian sperm movement
Source: Nat Commun. 2021 Jun 21;12:3808. doi: 10.1038/s41467-021-24011-0 (PMC8217517; doi:10.1038/s41467-021-24011-0)
Supplement: Supplementary file 3 — Description of Additional Supplementary Files [file 41467_2021_24011_MOESM3_ESM.pdf]

Description of Additional Supplementary Files

File Name: Supplementary Movie 1: Movie depicting STORM images in 3D rotation.
Movie showing 3D rotation of low magnification sperm cell with FAM161A staining.

File Name: Supplementary Movie 2: Movie depicting STORM images in 3D rotation.
Movie showing 3D rotation with zoom in at DC with FAM161A staining.

File Name: Supplementary Movie 3: Movie depicting STORM images in 3D rotation.
Movie showing 3D rotation of low magnification sperm cell with tubulin staining.

File Name: Supplementary Movie 4: Movie depicting STORM images in 3D rotation.
Movie showing 3D rotation with zoom in at sperm cell's DC with tubulin staining.

File Name: Supplementary Movie 5: Movie depicting rod movement.
A succession of images to reconstruct the DC motion inner scaffold as labeled by FAM161A as a proxy to model DC motion during flagella beating.

File Name: Supplementary Movie 6: Movie depicting modeling of straight, mild left, and sharp left cells, based on Cryo-ET imaging, as shown in Fig 4c.

Segmented columns are shown in grey, the proximal centriole (PC) in yellow, distal centriole (DC) doublets in green, distal centriole singlets in pink, the bars in light yellow. The cryo-ET Movie provides three main insights: 1) movement of the segmented columns, (2) movement of the PC relative to the DC, the PC to tilt towards the DC, and to shift laterally towards the DC centerline. (3) Movement (or rather lack thereof) of the bars. Note the Movie is for illustration purposes only as it comes from three different cells and not capturing one cell's movement. Tilting deformation of the connecting piece as well as the upward sliding of the right-segmented columns.

File Name: Supplementary Movie 7: Movie depicting modeling of straight, mild left, and sharp left cells, based on Cryo-ET imaging, as shown in Fig 4c.

Segmented columns are shown in transparent, the proximal centriole (PC) in yellow, distal centriole (DC) doublets in green, distal centriole singlets in pink, the bars in light yellow. The cryo-ET Movie provides three main insights: 1) movement of the segmented columns, (2) movement of the PC relative to the DC, the PC to tilt towards the DC, and to shift laterally towards the DC centerline. (3) Movement (or rather lack thereof) of the bars. Note the Movie is for illustration purposes only as it comes from three different cells and not capturing one cell's movement. Tilting deformation of the connecting piece as well as the upward sliding of the right-segmented columns.

File Name: Supplementary Movie 8: Movie depicting putative tail and head bending in sharp left to slight right sperm cells.

The still images aligned at the straight neck were played as a Movie to depict the head and tail bending from sharp left to slight right cells based on 3D-STORM and tubulin staining. Multiple cells were selected based on the curvature (higher to lower for sharp left – straight and lower to higher for slight right cells) from each group. The head is outlined with a white line, and the dotted orange line depicts the neck midline. Scale bar 2 μm.
