# Supplementary figures and images for: A dynamic basal complex modulates mammalian sperm movement
Source: Nat Commun. 2021 Jun 21;12:3808. doi: 10.1038/s41467-021-24011-0 (PMC8217517; doi:10.1038/s41467-021-24011-0)

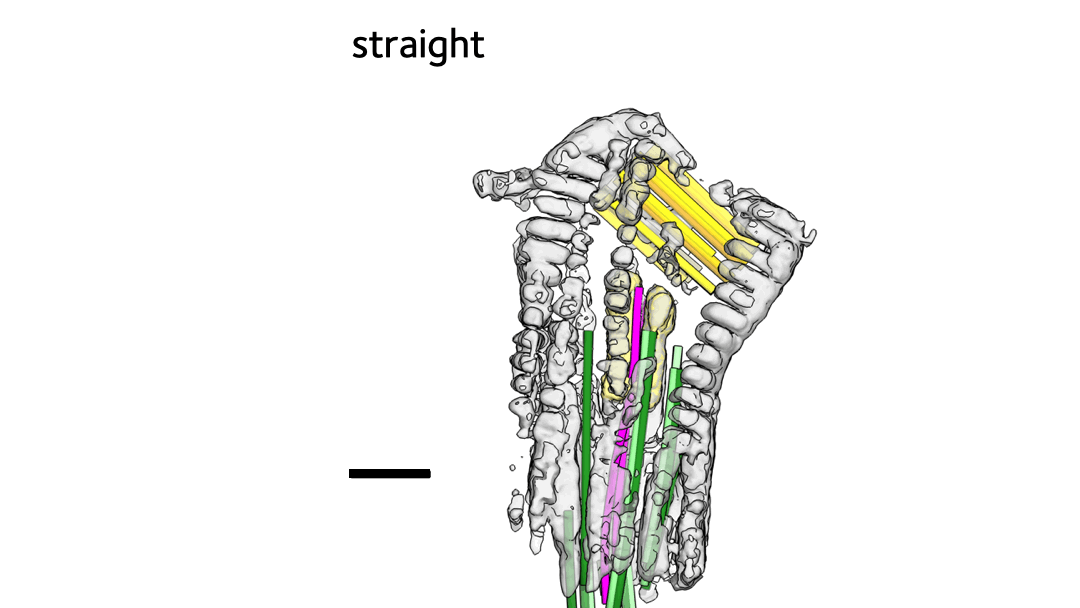

Supplement: Supplementary file 9 — Supplementary Movie 6 [file 41467_2021_24011_MOESM9_ESM.gif]

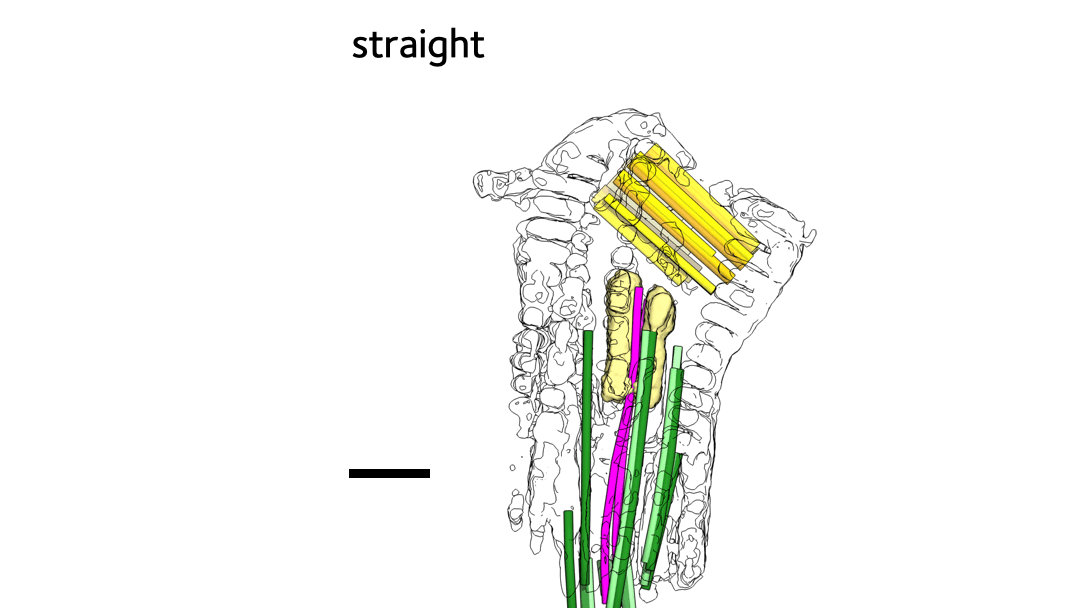

Supplement: Supplementary file 10 — Supplementary Movie 7 [file 41467_2021_24011_MOESM10_ESM.gif]
