# Supplementary material for: A dynamic basal complex modulates mammalian sperm movement
Source: Nat Commun. 2021 Jun 21;12:3808. doi: 10.1038/s41467-021-24011-0 (PMC8217517; doi:10.1038/s41467-021-24011-0)
Supplement: Supplementary file 12 — Reporting Summary [file 41467_2021_24011_MOESM12_ESM.pdf]

nature research

Corresponding author(s): Avidor-Reiss Tomer

Last updated by author(s): 2021 02 01

Reporting Summary

Nature Research wishes to improve the reproducibility of the work that we publish. This form provides structure for consistency and transparency in reporting. For further information on Nature Research policies, see our Editorial Policies and the Editorial Policy Checklist.

Statistics

For all statistical analyses, confirm that the following items are present in the figure legend, table legend, main text, or Methods section.

n/a	Confirmed	
☐	☒	The exact sample size (*n*) for each experimental group/condition, given as a discrete number and unit of measurement
☐	☒	A statement on whether measurements were taken from distinct samples or whether the same sample was measured repeatedly
☐	☒	The statistical test(s) used AND whether they are one- or two-sided<br>*Only common tests should be described solely by name; describe more complex techniques in the Methods section.*
☐	☒	A description of all covariates tested
☐	☒	A description of any assumptions or corrections, such as tests of normality and adjustment for multiple comparisons
☐	☒	A full description of the statistical parameters including central tendency (e.g. means) or other basic estimates (e.g. regression coefficient) AND variation (e.g. standard deviation) or associated estimates of uncertainty (e.g. confidence intervals)
☐	☒	For null hypothesis testing, the test statistic (e.g. *F*, *t*, *r*) with confidence intervals, effect sizes, degrees of freedom and *P* value noted<br>*Give P values as exact values whenever suitable.*
☒	☐	For Bayesian analysis, information on the choice of priors and Markov chain Monte Carlo settings
☒	☐	For hierarchical and complex designs, identification of the appropriate level for tests and full reporting of outcomes
☐	☒	Estimates of effect sizes (e.g. Cohen's *d*, Pearson's *r*), indicating how they were calculated

Our web collection on statistics for biologists contains articles on many of the points above.

Software and code

Policy information about availability of computer code

Data collection	Cryo-ET data was collected semi-automatically with SerialEM v3.8 (Mastronarde, 2005) and pre-processed on the fly with Warp v1.0 (Tegunov and Cramer, 2019).<br>Confocal data was collected on Leica SP8 confocal microscope. STORM data was collected on Nikon N-STORM4.0 system using an Eclipse Ti inverted microscope, an Apo TIRF 100X SA NA 1.49 Plan Apo oil objective, and a back-illuminated EMCCD camera (Andor, DU897).
Data analysis	Matlab R2020b was used for image processing, tail shape segmentation and subsequent curve, flagellum and sliding filament analysis using a mixture of built-in functions and bespoke scripts as described in Supporting Information. IBM SPSS v 26 was used for Exploratory Factor Analysis (EFA). Cryo-ET Tilt series were aligned with IMOD v4.10.25 (Kremer et al, 1996). Tomograms were segmented both manually using IMOD v4.10.25 and semi-automatically using the neural network based implementation in EMAN v2.21 (Chen et al, 2017). All measurements were performed in IMOD v4.10.25 and Fiji v2.0 (Schindelin et al., 2012).<br>All the confocal images were processed and quantified by using LasX software. Some images were processed by using Leica Hyvolution system 2 system. All the 3D-STORM images were analyzed and quantified by using NIS Elements (Nikon). All the publication ready images were prepared in Adobe photoshop CS6 and Adobe Illustrator CS6. Graphs were prepared using Prism v8. Averages, standard deviation and t-test were performed in Microsoft Excel 2011.

For manuscripts utilizing custom algorithms or software that are central to the research but not yet described in published literature, software must be made available to editors and reviewers. We strongly encourage code deposition in a community repository (e.g. GitHub). See the Nature Research guidelines for submitting code & software for further information.

Data

Policy information about availability of data

All manuscripts must include a data availability statement. This statement should provide the following information, where applicable:

- Accession codes, unique identifiers, or web links for publicly available datasets
- A list of figures that have associated raw data
- A description of any restrictions on data availability

Relevant data supporting the findings in this study are available in this paper and supporting information file. Source data and associated measurements are provided with this paper. Data shown in this manuscript is available from the corresponding author with a reasonable request.

Field-specific reporting

Please select the one below that is the best fit for your research. If you are not sure, read the appropriate sections before making your selection.

☒ Life sciences ☐ Behavioural & social sciences ☐ Ecological, evolutionary & environmental sciences

For a reference copy of the document with all sections, see nature.com/documents/nr-reporting-summary-flat.pdf

Life sciences study design

All studies must disclose on these points even when the disclosure is negative.

Sample size	Sample sizes are noted in the figure legend or on the figures . The sample sizes were not predetermined by any statistical method. The sample size is the number of sperm analyzed. Comparable sample were chosen to allow reproducibility for each experimental condition. Confocal data was collected from at least 3 experimental repetitions with each condition. Sample size ranges 47- 65 sperm cells depending upon the species and protein analyzed. The 3D-STORM data was also collected from at least 3 repetitions with each condition and sample size rages 15-97 sperm cells depending on experiment and protein labeled with. Cryo-ET Data was collected on a two (2) grids over a total of two (2) imaging sessions. A total of 27 sperm were analyzed. Data shown in the paper are cumulative of all repetitions.
Data exclusions	No data were excluded. All images were quantified for all the parameters as much as possible. Some of the parameters were not measured in some images because structures were not distinct. Cryo-Measurements were only performed on tomograms where microtubule-based structures were clearly visible.
Replication	All attempts of replication were successful. All Confocal and STORM experiments were repeated at least three times independently. The Cryo-ET imaging was repeated at least twice. Observations in all experiments were consistent. The final data pool together results from different experiments.
Randomization	We did not randomized the experiments since samples were prepared and analyzed in same time. To prevent bias, all the data in the field of view was analyzed.
Blinding	The researchers were not blinded for this study. They analysed each experiment knowing exactly which experiment was, since for most of the work the first author was the one performing experiments, preparing samples and analyzing them at the same time.

Reporting for specific materials, systems and methods

We require information from authors about some types of materials, experimental systems and methods used in many studies. Here, indicate whether each material, system or method listed is relevant to your study. If you are not sure if a list item applies to your research, read the appropriate section before selecting a response.

Materials & experimental systems

n/a	Involved in the study	
☐	☒	Antibodies
☒	☐	Eukaryotic cell lines
☒	☐	Palaeontology and archaeology
☒	☐	Animals and other organisms
☐	☒	Human research participants
☒	☐	Clinical data
☒	☐	Dual use research of concern

Methods

n/a	Involved in the study	
☒	☐	ChIP-seq
☒	☐	Flow cytometry
☒	☐	MRI-based neuroimaging

Antibodies

Antibodies used

Primary antibodies:
FAM161A : Sigma Aldrich, #HPA032119, Lot : A106842
WDR90 : Sigma Aldrich, #HPA061785, Lot : VF3017114A
POC5 : Thermo Fisher Scientific, # PA5-24308
POC1B :Thermo Fisher Scientific, # PA5-24495, Lot : UF2781655
CETN1 clone 2A6 : Santa Cruz, # sc-293494, Lot : L2717
Beta Tubulin (E7) : DSHB, AB_2315513
Secondary antibodies:
Anti rabbit Alexa647 : Jackson Immunoresearch, #711-605-152 , Lot : 210527
Anti Mouse Alexa647 : Jackson Immunoresearch, #715-605-150, Lot : 105758
Anti Mouse Alexa488 : Jackson Immunoresearch,# 715-545-150

Validation

FAM161A is prestige antibody with enhanced validation to ensure the specificity. All the validation data can be found in company's website. We also validated this antibody by expressing FAM161A in U2OS cell and labeling it with the antibody. In addition, we tested this antibody in Human, Bovine, and Rabbit sperm cell by labeling two centrioles. This antibody also has been used in other study PMID: 24833722.

WDR90 antibody has been validated by company and it has also been other study, PMID: 32946374. In addition, we tested this antibody in Human, Bovine, and Rabbit sperm cell by labeling two centrioles.

POC5 antibody is validated by company. We also validated this antibody by expressing POC5 in U2OS cells and labeling it by this antibody. In addition we checked this antibody in Human, and bovine sperm cells. It was specifically labeling two centrioles at sperm's neck. This antibody also has been used in other study, PMID: 29880810.

POC1B antibody has been used in other studies, PMID: 27185865, PMID: 30168418. In addition, we tested this antibody in Human, Bovine, and Rabbit sperm cell by labeling two centrioles.

CETN1 (2A6) antibody has been used in other study, PMID: 29880810. In addition, we tested this antibody in Human, Bovine, and Rabbit sperm cell by labeling two centrioles.

Beta Tubulin (E7) antibody has been used in many other studies (PMID: 27678524 and PMID: 26287727). In addition, we tested this antibody in Human, Bovine, and Rabbit sperm cell by labeling two centrioles.

The secondary anybodies, Anti rabbit A647, anti mouse A647, and anti mouse A488 are validated by company. We also validated them for their specificity by using them in negative control.

Human research participants

Policy information about studies involving human research participants

Population characteristics

Ejaculated spermatozoa were purchased from Manhattan Cryobank and Fairfax Cryobank (human). Human sperm samples were acquired with approval from the University of Toledo's Institutional Review Board (IRB).
Donor details for human spermatozoa purchased
Donor ID : 2148 Blood type : O+ CMV antibody : Negative

Genetic test	Result
Cystic Fibrosis-NonJ	Acceptable
Hemoglobin Variant	Acceptable
Karyotype	Acceptable
Cystic Fibrosis 86 mutation	Acceptable
Sickle Cell	Acceptable
Alpha-1 Antitrypsin	Acceptable
Blood group and Rh	Acceptable

Recruitment

We analyzed sperm sample from single fertile men, which may not be representative of whole population.

Ethics oversight

University of Toledo's Institutional Review Board (IRB).

Note that full information on the approval of the study protocol must also be provided in the manuscript.
